# Synergetic Effect of Hybrid Conductive Additives for High-Capacity and Excellent Cyclability in Si Anodes

**DOI:** 10.3390/nano12193354

**Published:** 2022-09-26

**Authors:** Byeong-Il Yoo, Han-Min Kim, Min-Jae Choi, Jung-Keun Yoo

**Affiliations:** 1 Carbon Composites Department, Composites Research Division, Korea Institute of Materials Science (KIMS), Changwon 51508, Korea; 2 Department of Chemical and Biochemical Engineering, Dongguk University, Seoul 04620, Korea; 3 Advanced Materials Engineering Division, University of Science and Technology (UST), Daejeon 34113, Korea

**Keywords:** lithium-ion batteries, conductive additive, carbon black, carbon nanotubes

## Abstract

Silicon is a promising anode material that can increase the theoretical capacity of lithium-ion batteries (LIBs). However, the volume expansion of silicon remains a challenge. In this study, we employed a novel combination of conductive additives to effectively suppress the volume expansion of Si during charging/discharging cycles. Rather than carbon black (CB), which is commonly used in SiO anodes, we introduced single-walled carbon nanotubes (SWCNTs) as a conductive additive. Owing to their high aspect ratio, CNTs enable effective connection of SiO particles, leading to stable electrochemical operation to prevent volume expansion. In addition, we explored a combination of CB and SWCNTs, with results showing a synergetic effect compared to a single-component of SWCNTs, as small-sized CB particles can enhance the interface contact between the conductive additive and SiO particles, whereas SWCNTs have limited contact points. With this hybrid conductive additive, we achieved a stable operation of full-cell LIBs for more than 200 cycles, with a retention rate of 91.1%, whereas conventional CB showed a 74.0% specific capacity retention rate.

## 1. Introduction

The energy density of lithium-ion batteries (LIBs) has become an important issue that needs to be addressed. For high-energy and high-density LIBs, it is important to use a high-capacity anode and active cathode materials [1,2]. Commercialized LIBs employ Ni-rich NCM (LiNi_x_Co_y_Mn_z_O_2_ (x + y + z = 1), with a theoretical specific capacity of 278 mAh/g) as cathode material and graphite (theoretical specific capacity of 372 mAh/g) blended with silicon (Li_15_Si_4_, theoretical specific capacity of 3578 mAh/g) as anode materials [3,4,5,6]. To obtain high-capacity anodes, it is necessary to increase the ratio of the Si component relative to graphite. The addition of more than 10% Si in the anode could potentially achieve an anode with high specific capacity (>500 mAh/g, graphite 90% + Si 10%), decreasing the its thickness, in addition to improving output power [7,8]. However, it is still a considerable challenge to increase the proportion of Si components in anodes to more than 10% due to volume expansion of Si during lithiation; Si exhibits volume expansion and shrinkage during charging/discharging of Li-ions, which causes pulverization of Si particles, as well as isolation of particles [9,10]. In addition, the solid electrolyte interphase (SEI) layer produced during charging/discharging cycles is continuously fractured and reformed, leading to a lack of electrolytes and a decrease in anode capacity [11,12].

To solve this problem, various shapes and structures of active Si materials have been proposed, including nanosized Si [13,14], Si nanotubes [15,16], Si nanowire [17,18,19], hollow Si [20], and porous Si [21,22]. In particular, a specific size of Si particles (<150 nm) could effectively suppress pulverization and crack formation in response to volume expansion [23]. Nevertheless, Si nanoparticles have a high surface-area-to-volume ratio, making it difficult to achieve homogeneous dispersion in slurry and resulting in a thick SEI layer during the first cycle of charging and discharging, which causes high irreversible capacity loss. Recently, binders with novel functionality, such as strong adhesion force and self-healing, have also been reported for Si anodes [24,25,26,27,28,29]. However, studies on the effect of conductive additives in Si anodes have not been conducted to date.

**Figure 1:**
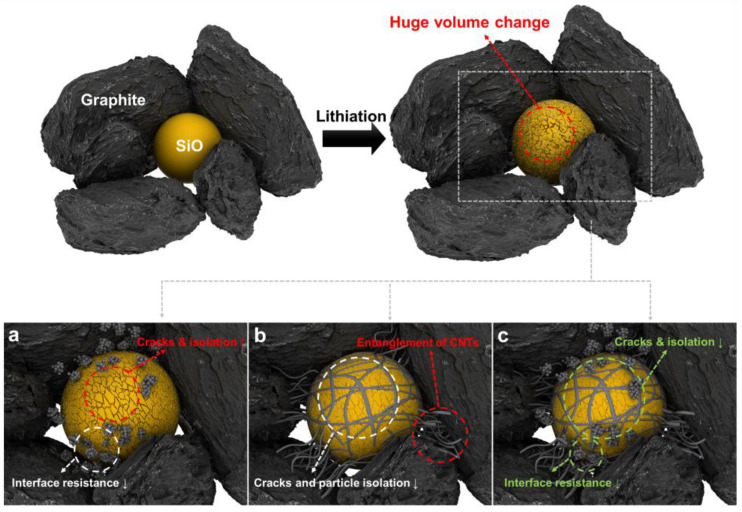
Schematic illustration of an electrode using (**a**) CB, (**b**) SWCNTs, and (**c**) hybrid additive.

Here, we propose that well-combined conductive additives can dramatically improve the performance and cyclability of Si anodes. We hypothesize that carbon black (CB), a conventional conductive additive, cannot connect Si particles after pulverization (Figure 1). Instead, we introduce single-walled carbon nanotubes (SWCNTs) as a conductive additive to effectively connect Si particles, enabling not only suppression of volume expansion of Si particles but also an electrical network of fractured Si particles, owing to the high aspect ratio of SWCNTs. We further report that the mixture of CB and SWCNTs has a synergetic effect: CB improves the interface resistivity between the conductive additive and Si particles, and SWCNT crosslinks the Si particles. Consequently, hybrid conductive additives in Si anodes (7 mg/cm^2^ by 97% active material) exhibit an excellent cyclability of more than 200 cycles, with a retention rate of 91.1% and a superior retention rate of 92.6% in a fast charge/discharge test at a current rate of 4 C. Furthermore, we find that, with hybrid conductive additives, a 50% reduction in the amount of conductive additives achieves similar electrochemical performance in full-cell LIBs, suggesting that even a small amount of additional SWCNTs (0.05 wt%) could dramatically improve the overall electrochemical stability of Si anodes.

## 2. Materials and Methods

### 2.1. Materials and Electrode Preparation

CB powder (Super C65) and SWCNT dispersion were purchased from Imerys Co., Ltd. (Paris, France.), and Advanced Nano products Co., Ltd. (Sejong, Korea), respectively. Graphite (BTR Co., Ltd., Shenzhen, China) and SiO (Osaka Titanium Co., Ltd., Osaka, Japan.) were used as received without further processing. Styrene butadiene rubber (SBR) solution was purchased from JSR Co., Ltd. (Tokyo, Japan), and Carboxymethyl cellulose (CMC) powder was purchased from Daicel Co., Ltd. (Himeji-shi, Japan). The CMC powder was dissolved (1.5 wt%) in deionized water.

The anode slurry consisted of natural graphite and SiO (95:5, weight ratio) as active material, with SBR (1.7 wt%) and CMC (0.8 wt%) as a binder. CB and SWCNTs were added as conductive materials. The contents of each sample are listed in Table 1.

All the slurries were mixed for 15 min using a planetary centrifugal mixer at 1300 rpm. The slurries were blade-coated onto 18 µm thick copper foil and dried at 120 °C in a vacuum for 12 h. The mass loading of the electrode was approximately ~7 mg/cm^2^. The cathode used as counter electrode was prepared by coating N-methyl-2-pyrrolidone (NMP) (Daejung Chemical & Materials Co., Ltd., Siheung, Korea) base slurry onto 20 µm thick Al foil. The cathode slurry consisted 97.6 wt% LiNi_0.8_Co_0.1_Mn_0.1_O_2_ (Hunan Shanshan Toda Advanced Materials Co., Ltd., Changsha, China) as active material. Then, 0.9 wt% multiwalled CNT (Advanced Nano products Co., Ltd., Sejong, Korea) and 0.8 wt% polyvinylidene fluoride (PVDF) (Kureha Co., Ltd., Tokyo, Japan) were added as conductive additives and binder, respectively.

### 2.2. Materials Characterization

The swelling ratio was measured with a μ-HITE instrument (TESA). A 2 cm × 2 cm electrode was immersed in electrolytes for 24 h, and the thickness was measured again. The average value of 9 points was used for the thickness, and all wetting was conducted in a glove box under a pure Ar atmosphere.

SEM images were observed using a JSM-6700F instrument (JEOL, Tokyo, Japan) under an accelerating voltage of 15 kV. The electrodes were observed after treatment with cross-section polisher using an IB-19520CCP instrument (JEOL) under an accelerating voltage of 4 kV to obtain cross-sectional SEM images.

### 2.3. Electrochemical Characterization

The resistances of the electrodes were measured using a Hioki electrode resistance meter (XF-057) with constant current (10 mA). To evaluate the electrochemical performance of the electrodes, coin cells (2032 coin) consisting of an anode electrode, a polypropylene (PP) separator (Celgard 2400), electrolytes (1 M LiPF_6_ in EC:DEC (ethylene carbonate:diethyl carbonate), 3:7 + 5 wt% fluoroethylene carbonate (FEC)), and an Li counter electrode or an NCM811 cathode electrode were assembled in a glove box under highly pure Ar. The punched electrode diameters were as follows: anode: 14 mm for half cell,16 mm for full cell; cathode: 14 mm for full cell. The full cells were designed with an N/P ratio (areal capacity ratio of anode-to-cathode) of 1.1.

Galvanostatic charge/discharge tests for the anode half cell were performed in the voltage range of 0.05–1.5 V at 45 °C were at 0.5 C. Galvanostatic charge/discharge tests for the anode full cells were performed in the voltage range of 2.8–4.25 V at 25 °C at various C rates (C/10, C/5, C/2, 1 C, 2 C, 3 C, and 4 C). Charge–discharge curves were recorded using a WBCS 3000 battery tester system (WonA Tech). Electrochemical impedance spectroscopic analysis was performed at frequencies of 0.1 MHz to 0.1 Hz with a voltage amplitude of 0.01 V using a ZIVE SP1 electrochemical workstation (WonATech, Seoul, Korea). In addition, Galvanostatic intermittent titration technique (GITT) measurements of cells were performed at 0.5 C delivered a current pulse for 5 min in the voltage range of 2.8–4.25 V, and direct current internal resistance (DCIR) measurements were performed using coin cells in the voltage range of 2.8–4.25 V.

## 3. Results and Discussion

**Figure 2:**
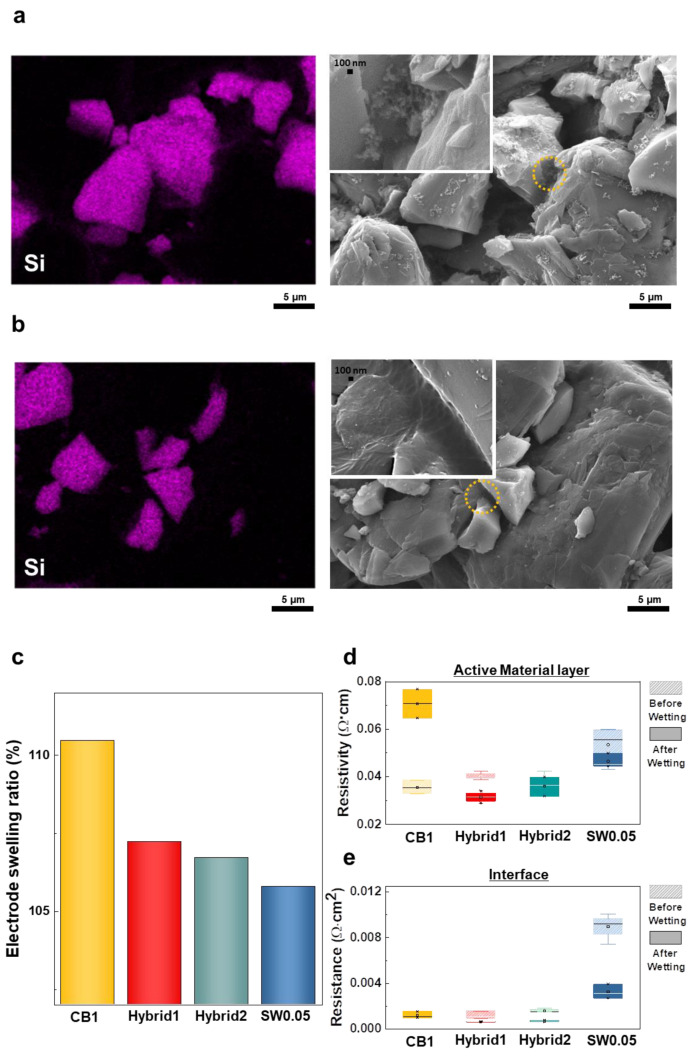
Characterization of electrodes: EDS and SEM images of electrode surfaces using (**a**) CB1 and (**b**) SW0.05. (**c**) Electrode swelling ratio after electrolyte wetting. (**d**) Change in active material resistivity after electrolyte wetting. (**e**) Change in interface resistance after electrolyte wetting.

We began by preparing anode electrodes using different conductive additives (i.e., CB and SWCNTs). To observe differences in morphology, we obtained surface SEM images of the electrodes. When CB was used as a conductive additive, we observed local aggregation of CB particles, as shown in Figure 2a. In the case of SWCNT additive, the SWCNTs exhibited interconnection through the active SiO particles (Figure 2b).

To understand the interconnection of SWCNTs in the electrode, we measured volume expansion and resistivity changes of the electrodes after wetting with electrolytes. We prepared four electrodes with the following conductive additives: 1 wt% CB (CB1); mixture of 0.95 wt% CB and 0.05 wt% SWCNTs (Hybrid1); mixture of 0.45 wt% CB and 0.05 wt% SWCNT (Hybrid2); and 0.05 wt% SWCNT (SW0.05). Figure 2c shows the swelling ratio of each electrode after 24 h of wetting with electrolytes. CB1 showed the highest swelling ratio of ~110%, whereas the electrode containing SWCNTs had a much lower swelling ratio, implying that the addition of SWCNTs to the electrode hinders volume expansion of electrode through its network structure.

We further measured the resistivity of electrodes after swelling with electrolytes to determine an effect of volume expansion in terms of electrical properties. Table 2 summarizes the resistivity and resistance of each electrode. The CB1 showed a twofold increase in resistivity after wetting, and the electrode containing SWCNTs showed similar resistivity after wetting (Figure 2d), which could be explained by the fact that SWCNTs produce a network between SiO particles, causing no change in resistivity with increased physical distance between SiO particles. In contrast, the small particle size of CB could did not induce contact between SiO particles with increased physical distance. As a result, CB1 presented with the highest resistivity after wetting. Interface resistance refers to the resistance between the current collector and the active material layer. When contact is poor, interface resistance increases, accounting for a large part of the total electrode impedance [30]. CB1 showed much lower values than SW0.05, suggesting that CB enables better interface contact compared to SWCNTs (Figure 2e). When CB and SWCNTs were added in combination, the interface contact was considerably improved, as confirmed by the fact that Hybrid1 and Hybrid2 showed similarly low interface resistances compared to that of CB1.

**Figure 3:**
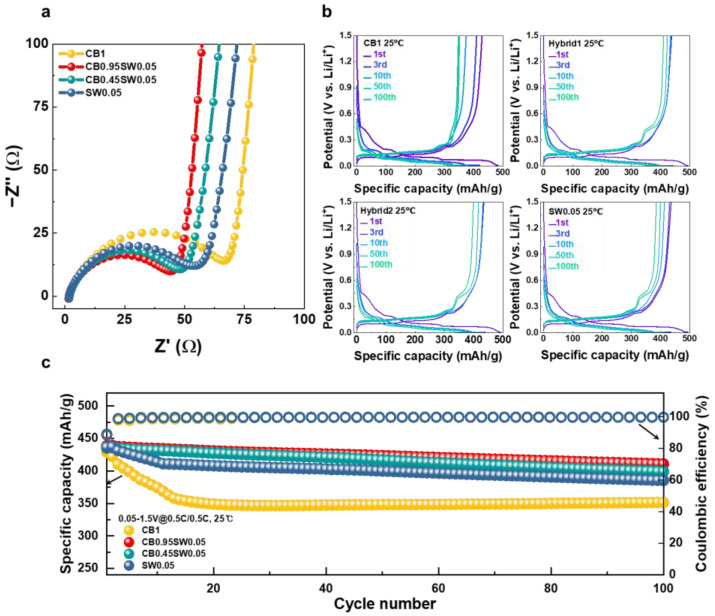
Characterization of electrochemical properties of half cells: (**a**) impedance measurement (EIS); (**b**) charge/Discharge curves in the 1st, 2nd, 3rd, 10th, 50th, and 100th cycles; and (**c**) cycle performance during 100 cycles.

We determined the electrochemical properties of anode electrodes by half-cell measurements. Electrochemical impedance spectroscopy (EIS) of half cells, as shown in Figure 3a and Table S1, indicated that the mixture of CB and SWCNTs (Hybrid1 and Hybrid2) exhibited lower resistivity against charge transport compared with the single-component conductive additive (CB1, SW0.05). The SW0.05 showed a lower R_ct_ value than CB1, indicating improved carrier conductivity of SWCNTs relative to that of CB [31,32,33].

Figure 3b,c describes the electrochemical performance and cyclability of anode half-cells at a current rate of 0.5 C in the 0.05–1.5 V window. The Hybrid1 electrode presented with excellent cyclability retaining 93.9% of the initial specific discharge capacity after 100 cycles. The other electrodes, including Hybrid2, SW0.05, and CB1 showed retention rates of 91.8%, 89.3%, and 85.7%, respectively, after 100 cycles. In the profiles of Hybrid 1, 2 and SW0.05, a plateau can be observed in the 0.4–0.45 V region, occurring during the delithiation of Li_15_Si_4_ [34]. The reason why such a plateau was not observed during 1st to 10th cycles is that SiO formed an oxide matrix (Li-Si-O). Li-Si-O inhibits the volume expansion of Si particles but still generates volume expansion of 200%, which causes strong compression stress [35]. This induced stress inhibits the formation of Li_15_Si_4_, so a plateau was not observed [36]. In contrast, no plateau was observed in any sections of CB1. In Figure 3c, the 10th cycle specific capacity of CB1 is 374.31 mAh/g. Given that the theoretical specific capacity of graphite is 372 mAh/g, Si particles did not play a role in the electrodes, suggesting that CB cannot suppressed particle pulverization and isolation. This can also be confirmed in terms of Coulombic efficiency (Figure S1). The coulombic efficiency of Hybrids 1 and 2, as well as that of SW0.05, gradually stabilized, whereas the CB1 battery showed unstable coulombic efficiency up to the 20th cycle as a result of continuous particle cracking.

**Figure 4:**
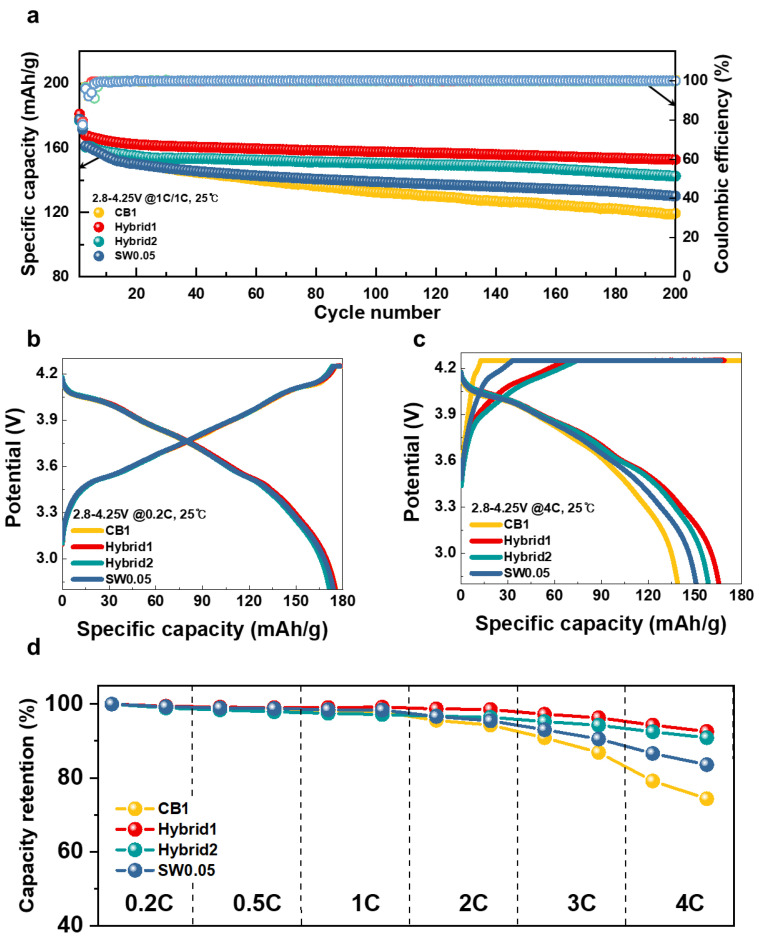
Characterization of electrochemical properties of full cells: (**a**) cycle performance during 200 cycles; (**b**) charge/discharge curves at 0.2 C; (**c**) charge/discharge curves at 4 C; and (**d**) rate cycling performance from 0.2 C to 4 C.

These anode electrodes were further employed in full-cell lithium-ion batteries (LIBs) to verify the electrochemical effect of SWCNTs. Figure 4a shows the cyclability of full cells at a current rate of 1 C in the 2.8–4.25 V window. Similarly to half-cell results, Hybrid1 showed the most stable cyclability, exhibiting 91.1% retention of specific discharge capacity after 200 cycles. Hybrid2, SW0.05, and CB1 displayed a retention of 88.7%, 80.7%, and 74.0%, respectively. The power capability at charging and discharging current rates of 0.2 C, 0.5 C, 1 C, 2 C, 3 C, and 4 C is shown in Figure 4b–d. Under the 0.2 C condition, there were no obvious differences between the electrodes (Figure 4b). The difference in charging/discharging profile increased with increased current rate, with a remarkable distinction at a fast charging/discharging rate of 4 C (Figure 4c) (see all charging/discharging profiles in Figure S3). Hybrid1, Hybrid2, SW0.05, and CB1 showed specific capacity retention rates of 92.6%, 90.9%, 83,6%, and 74.4%, respectively, under the 4 C condition. Figure 4d summarizes the specific capacity retention depending on current rate of the tested electrodes, in agreement with the results of the half-cell test, showing that a mixture of CB and SWCNTs in the electrode results in more stable electrochemical performance, whereas using CB resulted in rapid degradation in specific capacity retention. We suggest that CB cannot connect the active materials in the volume expansion during the charging and discharging cycle. In contrast, SWCNTs induced a conductive network between active materials, owing to the high aspect ratio retained by the conductive pathway with volume expansion. The poor interface contact of SWCNTs was improved by the addition of CB; thus, Hybrid1 and Hybrid2 resulted in the most stable electrochemical performance in both half-cell and full-cell measurements.

**Figure 5:**
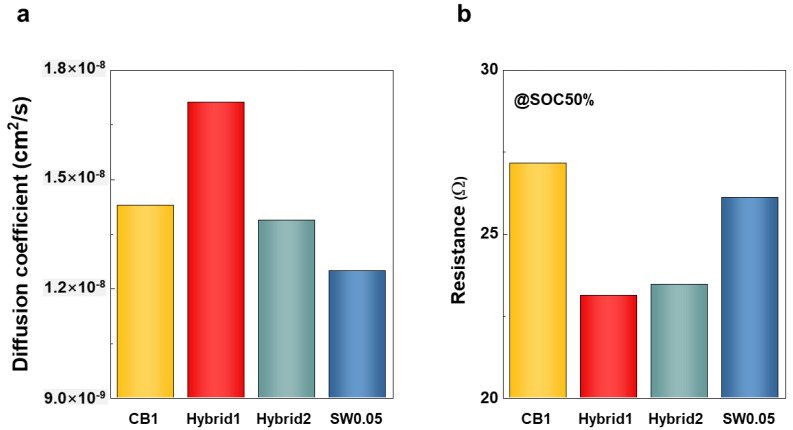
Characterization of electrochemical properties of cycled full cells: (**a**) Li-ion diffusion coefficient calculated using GITT at 0.5 C; (**b**) DCIR-based resistance at SOC50%.

To understand difference in electrochemical stability, we carried out GITT analyses on resistivity within the LIBs (see Figure S4 for full scan of GITT). The diffusion coefficient of Li-ions was calculated using Equation (1):

(1) $$D=\frac{4}{\pi \tau }{\left(\frac{n_{m}V_{m}}{S}\right)}^{2}{\left(\frac{\Delta E_{s}}{\Delta E_{t}}\right)}^{2}$$

where τ, S, n_m_, V_m_, ΔE_s_, and ΔE_t_ are the duration of the current pulse, electrode/electrolyte contact area, mole number, molar volume of the electrode, steady-state voltage changes according to current pulse, and voltage change during the constant current pulse, respectively [37]. As shown in Figure 5a and Table S3, the Li-ion diffusion coefficient of Hybrid1 was higher than that of CB1, in agreement with the electrochemical performance results. CB1 exhibited a higher diffusion coefficient of Li-ion than Hybrid2 and SW0.05. However, the absolute amount of conductive additive in Hybrid2 and SW0.05 was much lower than that in CB1. Considering the loading amounts of conductive additive, SWCNTs lead to more efficient Li-ion diffusion compared with CB.

Figure 5b describes the resistivity depending on state of charge (SOC) according to DCIR measurements. A current rate of 3 C was injected for 30 s depending on SOC (base current rate of 0.5 C) [38]. All electrodes presented with parabola curves, as shown in Figure S5. At an SOC of 50%, Hybrid1 exhibited the lowest resistance, followed by Hybrid2, SW0.05, and CB1 (Figure 5b). A similar trend occurred in other SOC values, as summarized in Table S4. This result confirms that the mixture of SWCNT and CB enables a reduced resistance in the electrode as a result of efficient Li-ion diffusion.

**Figure 6:**
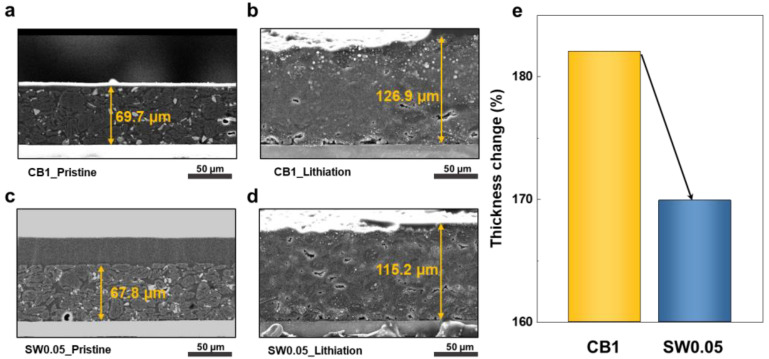
Characterization of cycled electrodes: cross-sectional SEM images of electrodes. Pristine electrodes with (**a**) CB 1 and (**c**) SW0.05 additives. Lithiation electrode with (**b**) CB1 and (**d**) SW0.05 additive. (**e**) Bar image of electrode thickness change.

To investigate structural changes in electrochemical operation, we obtained cross-sectional SEM images of the electrodes after lithiation (Figure 6a–d). Thickness changes after lithiation, as shown in Figure 6e, shows that SW0.05 exhibited less volume expansion compared to CB1, proving a more efficient suppression in volume expansion by SWCNTs than that induced by CB, with a smaller addition amount. This suggests that SWCNTs could be used as an efficient conductive additive to increase the energy density of LIB electrodes.

## 4. Conclusions

Here, we report a systematic study on the role of conductive additives in Si-based LIBs. CB additive resulted a low interface resistivity of the anode, owing the small particle size; however, it did not result in isolation of Si particles, leading to a decrease in capacity. On the other hand, SWCNTs, another conductive additive, suppressed the isolation of Si particles by crosslinking, although an increase in the amount of SWCNTs is accompanied by an increase in dispersants and solvents. As they inhibit energy density, the SWCNT content must be limited in order to achieve high energy density. We found that the introduction of a hybrid additive containing both CB and SWCNTs both suppressed volume expansion and minimized interface resistivity. Experimental GITT and DCIR measurements show that the hybrid conductive additive improved ion conductivity and electrical conductivity compared to single components (CB and SWCNTs). In addition, this hybrid conductive additive resulted in more stable cyclability of full-cell LIBs compared to the single-component additive. A 50% reduction in hybrid conductive additive (0.5 wt%) resulted in a similar output power and cyclability with LIBs using 1 wt% hybrid conductive additive. These results show that a hybrid conductive additive exerts a synergetic effect compared to single components, suggests a pathway for high-energy-density LIBs by reducing the portion of conductive additive.

## Figures and Tables

**Table 1:** Electrode compositions of four samples.

Sample Name	Graphite:SiO	CB	SWCNT	SBR	CMC
CB1	95:5	1 wt%	-	1.7 wt%	0.8 wt%
Hybrid1		0.95 wt%	0.05 wt%		
Hybrid2		0.45 wt%	0.05 wt%		
SW0.05		-	0.05 wt%		

**Table 2:** Resistivity and resistance of electrodes.

		CB1	Hybrid1	Hybrid2	SW0.05
Active Layer [Ω·cm]	Before Wetting	0.037	0.040	0.038	0.053
	After Wetting	0.071	0.035	0.039	0.050
Interface [Ω·cm^2^]	Before Wetting	0.0013	0.0012	0.0016	0.0090
	After Wetting	0.0014	0.0008	0.0009	0.0039

## Data Availability

Not applicable.

## Supplementary Materials

The following supporting information can be downloaded at: https://www.mdpi.com/article/10.3390/nano12193354/s1; Table S1: EIS fitting values in Figure 3a; Figure S1: Comparison of (a) 1st charge/discharge profiles (b) coulombic efficiency of the 4 samples half-cell; Figure S2: Comparison of (a) 1st charge/discharge profiles (b) coulombic efficiency of the 4 samples full-cell; Table S2: Initial coulombic efficiency values in Figure 3c and Figure 4a; Figure S3: Charge/discharge curves at various current density of (a) CB1 (b) Hybrid1 (c) Hybrid2 (d) SW0.05; Figure S4: (a) GITT curves for 4 samples. (b) Detailed GITT curves from 1200 to 2400 s at 0.5C; Table S3: Detailed values of Li ion diffusion coefficient; Figure S5: Resistance vs. SOC profiles during the charge; Table S4: Detailed values at various SOC.
