# Supplementary material for: Synergetic Effect of Hybrid Conductive Additives for High-Capacity and Excellent Cyclability in Si Anodes
Source: Nanomaterials (Basel). 2022 Sep 26;12(19):3354. doi: 10.3390/nano12193354 (PMC9565680; doi:10.3390/nano12193354)
Supplement: Supplementary file 1 [file nanomaterials-12-03354-s001.zip › nanomaterials-1894715-supplementary.pdf]

nanomaterials

MDPI

Supporting Information

Synergetic Effect of Hybrid Conductive Additives for High-Capacity and Excellent Cyclability in Si Anodes

Byeong-Il Yoo[1,2], Han-Min Kim[1,2], Min-Jae Choi[2,*], Jung-Keun Yoo[1,3,*]

1 Carbon Composites Department, Composites Research Division, Korea Institute of Materials Science (KIMS), Changwon 51508, Republic of Korea
2 Department of Chemical and Biochemical Engineering, Dongguk University, Seoul 04620, Republic of Korea
3 Advanced Materials Engineering Division, University of Science and Technology (UST), Daejeon, 34113, Republic of Korea
* Correspondence: minjae.choi@dgu.ac.kr (M.-J.C.) yoojk@kims.re.kr (J.-K.Y.)

Table.S1. EIS fitting values in Figure 3a.

EIS [Ω·cm]	CB1	Hybrid1	Hybrid2	SW0.05
R_s	1.92	1.74	1.99	1.84
R_{ct}	65.96	44.06	47.84	53.53

Figure.S1

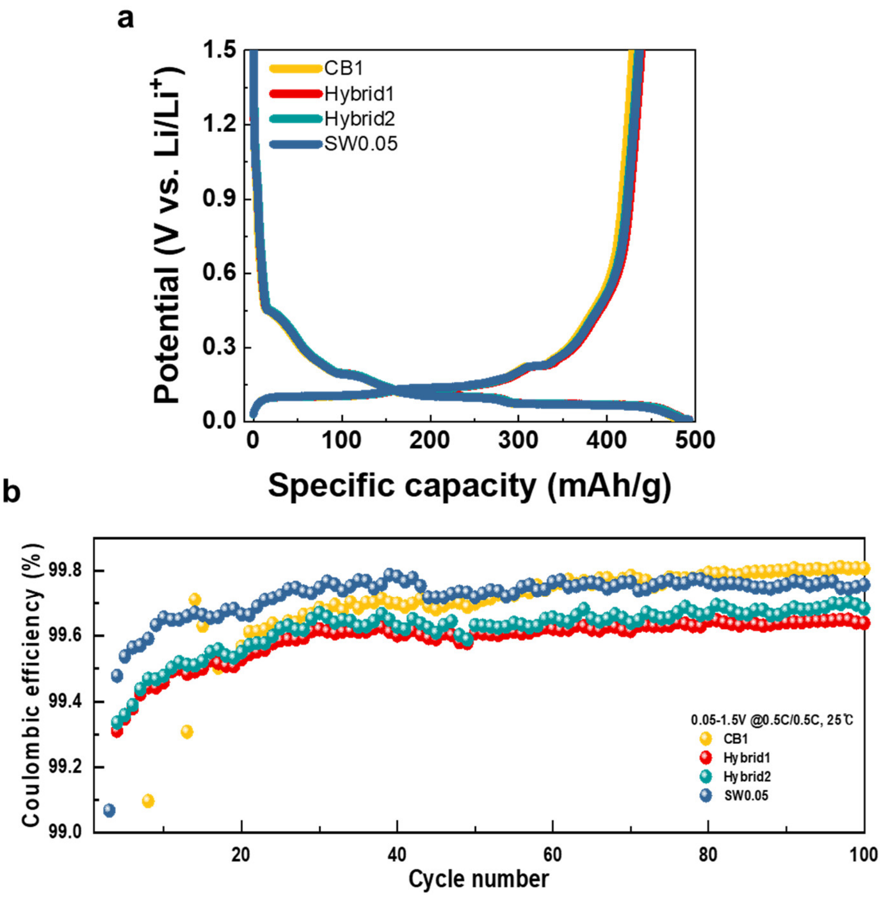

Figure.S1. Comparison of (a) 1st charge/discharge profiles (b) coulombic efficiency of the 4 samples half-cell.

Figure.S2

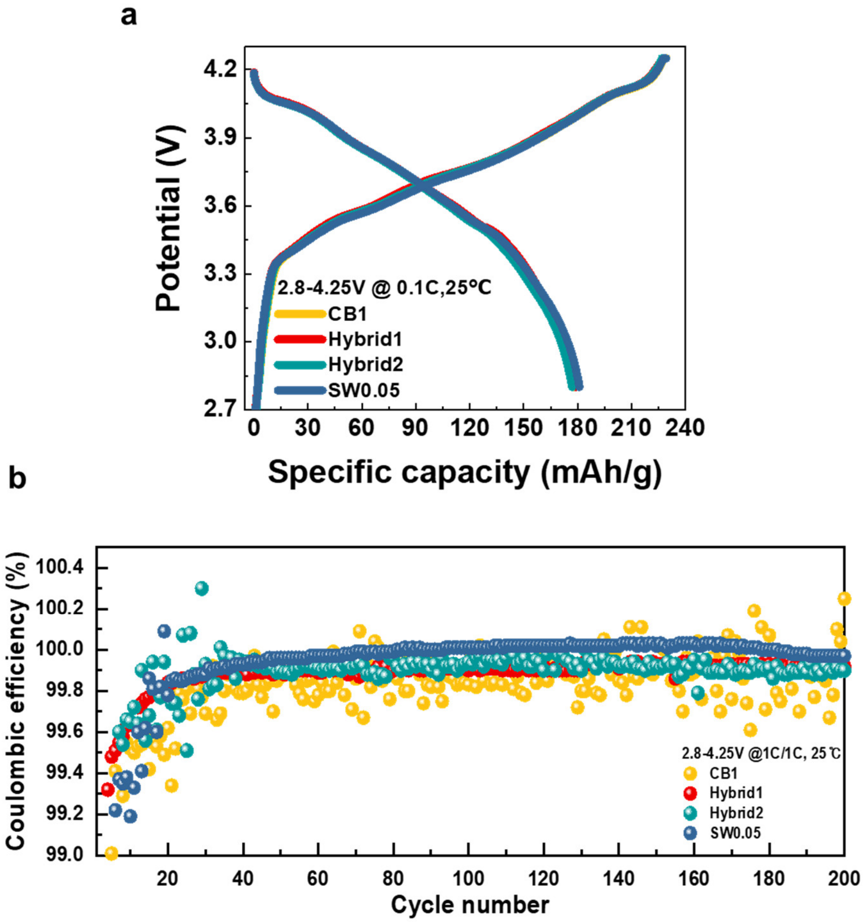

Figure.S2. Comparison of (a) 1st charge/discharge profiles (b) coulombic efficiency of the 4 samples full-cell.

Table.S2. Initial coulombic efficiency values in Figure 3c and Figure 4a.

		**CB1**	**Hybrid1**	**Hybrid2**	**SW0.05**
Initial Coulombic Efficiency [ICE]	Half-cell	88.71	88.92	88.37	88.60
	Full-cell	78.35	78.92	77.46	77.55

Figure.S3

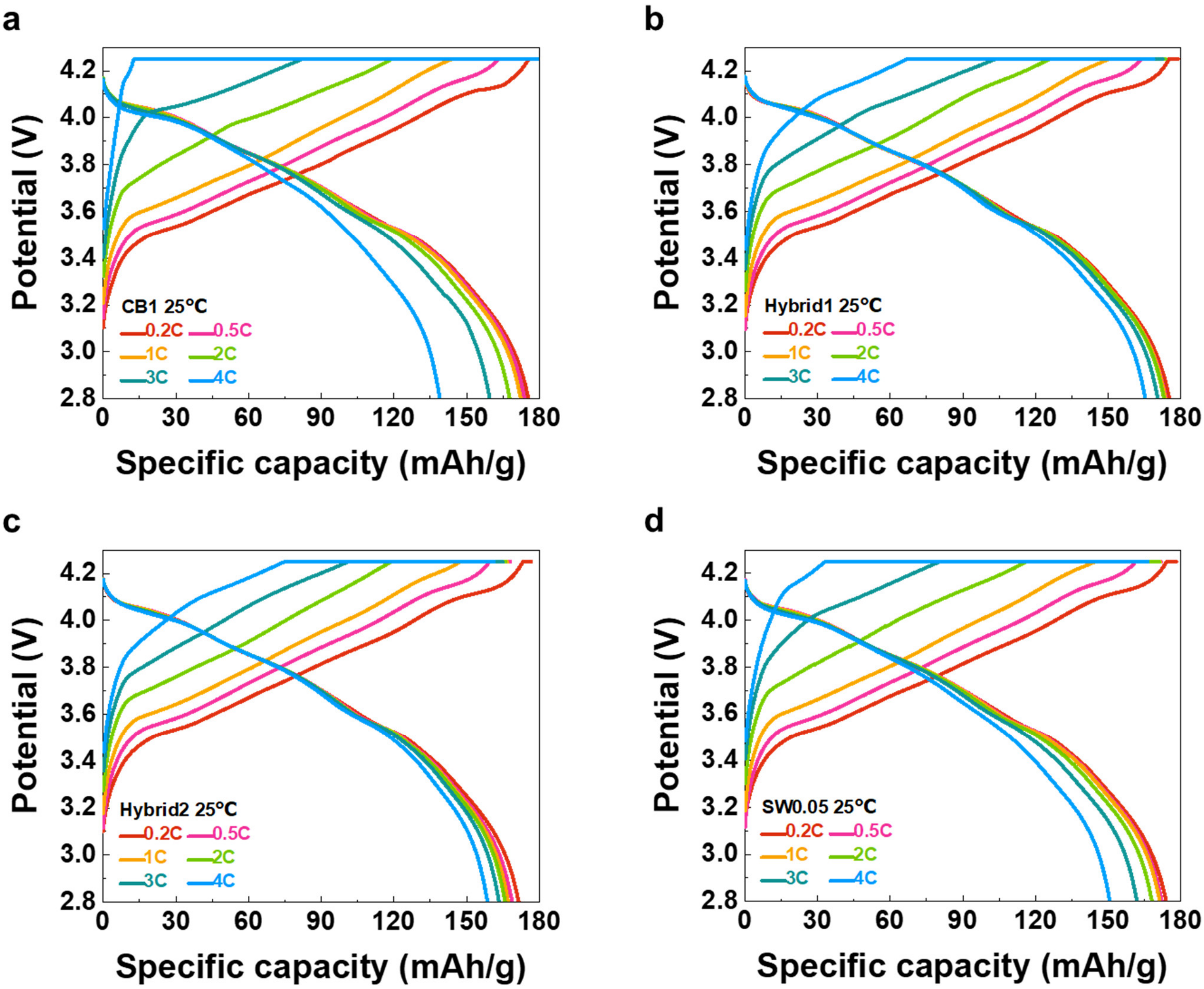

Figure.S3. Charge/discharge curves at various current density of (a) CB1 (b) Hybrid1 (c) Hybrid2 (d) SW0.05.

Figure.S4

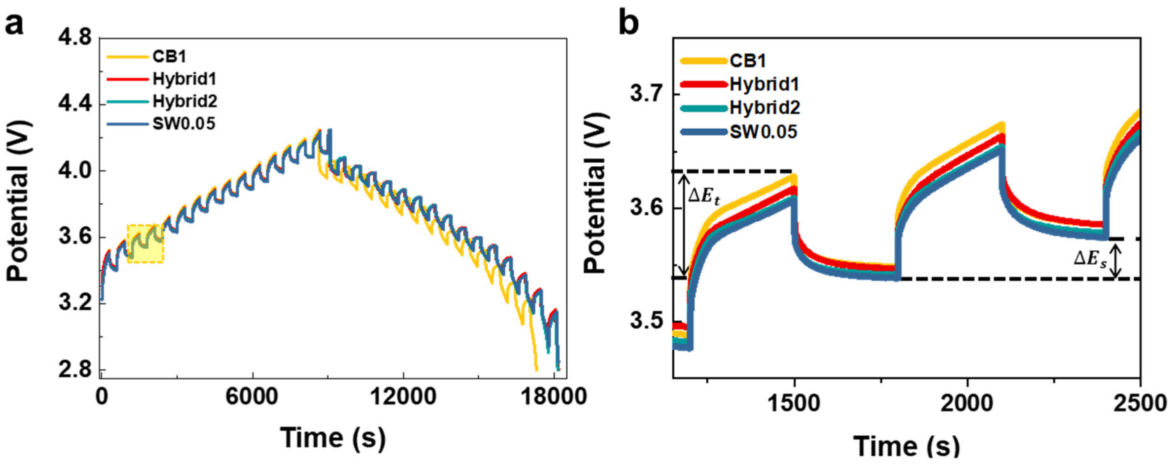

Figure.S4. (a) GITT curves for 4 samples. (b) Detailed GITT curves from 1200 to 2400 second at 0.5C.

Table.S3. Detailed values of Li ion diffusion coefficient.

	CB1	Hybrid1	Hybrid2	SW0.05
D_{Li^+} [cm^2/s]	1.43E-8	1.71E-8	1.39E-8	1.25E-8

Figure.S5

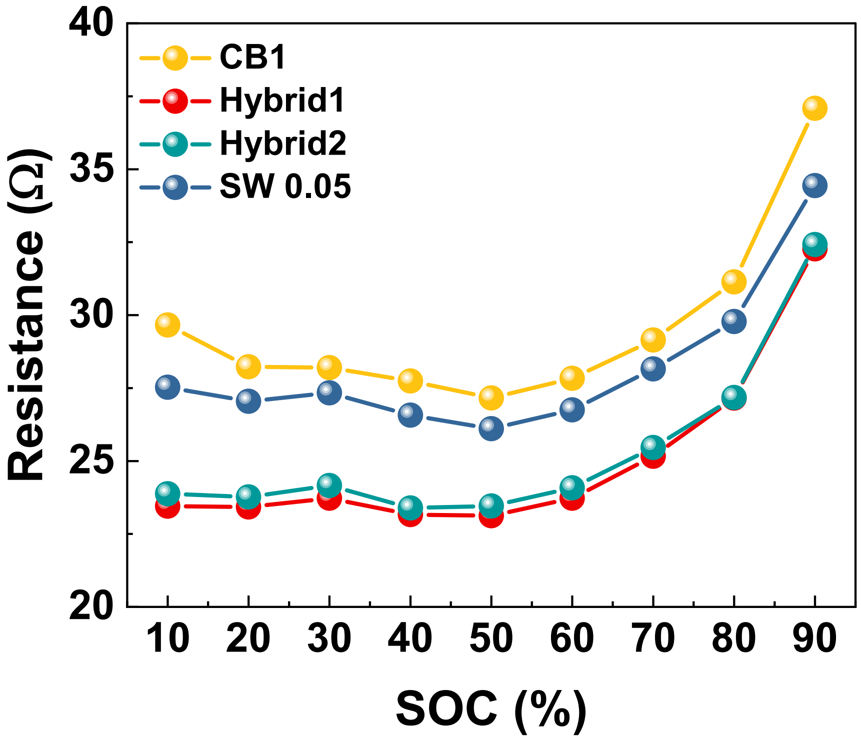

Figure.S5. Resistance vs SOC profiles during the charge.

Table.S4. Detailed values at various SOC.

IR [Ω]	CB1	Hybrid1	Hybrid2	SW0.05
@ SOC 10 %	29.66	23.45	23.88	27.52
@ SOC 50 %	27.16	23.12	23.46	26.11
@ SOC 90 %	37.09	32.27	32.41	34.43
